# Stiffness Changes in Shoulder Muscles between Pitchers and Position Players after Throwing Overhead Using Shear Wave Elastography and Throwing Motion Analysis

**DOI:** 10.3390/jcm13072056

**Published:** 2024-04-02

**Authors:** Hironori Tsurukami, Yoshiaki Itoigawa, Hirohisa Uehara, Fumitoshi Hatae, Atsushi Kubota, Motoki Mizuno, Katsuhiko Maezawa, Yuuji Takazawa, Muneaki Ishijima

**Affiliations:** 1 Department of Orthopaedic Surgery, Juntendo University Urayasu Hospital, Urayasu 279-0021, Japan; h-tsurukami@juntendo.ac.jp (H.T.); f-hatae@juntendo.ac.jp (F.H.); maeza@juntendo.ac.jp (K.M.); 2 Department of Orthopaedic Surgery, Faculty of Medicine, Juntendo University, Hongo, Tokyo 113-0034, Japan; hi-uehara@juntendo.ac.jp (H.U.); ishijima@juntendo.ac.jp (M.I.); 3 Graduate School of Health and Sports Science, Juntendo University, Inzai 270-1695, Japan; akubota@juntendo.ac.jp (A.K.); mtmizuno@juntendo.ac.jp (M.M.); takayuuji16@gmail.com (Y.T.); 4 Department of Sports Medicine, Faculty of Medicine, Juntendo University, Hongo, Tokyo 113-0034, Japan

**Keywords:** disabled throwing shoulder, shear wave elastography, throwing motion analysis, muscle strength, stiffness

## Abstract

**Objectives**: The objective is to compare stiffness changes around the shoulder muscles between pitchers and position players after throwing overhead using shear wave elastography (SWE) in relation to throwing motion analysis and muscle strength. **Methods**: A total of 32 male college baseball players (12 pitchers and 20 position players) were observed throwing 20 times, and SWE was performed to evaluate 13 shoulder muscle items—tendons (supraspinatus, infraspinatus, subscapularis, and teres minor), muscles (supraspinatus, infraspinatus [transverse and oblique part], teres minor, lower trapezius, latissimus dorsi, and pectoralis minor), and capsules (posterior and posteroinferior). Motion analysis was used to assess elbow torque, forearm angle, forearm rotation speed, and maximum external rotation angle of the shoulder. Muscle strength was measured using a dynamometer for abduction, internal/external rotation of the shoulder at an abduction of 0°, internal/external rotation of the shoulder at an abduction of 90°, and internal/external rotation of shoulder at a flexion of 90°. **Results**: In the pitcher group, SWE values for the teres minor muscle and latissimus dorsi muscle increased significantly after throwing. In the position player group, SWE values for the teres minor muscle significantly increased, and SWE values of the pectoralis minor muscle decreased after throwing. In the pitcher group, positive correlations were found between the teres minor muscle and forearm rotation speed and between the latissimus dorsi muscle and forearm angle. No significant difference was found in muscle strength after throwing in any of the groups. **Conclusions**: Stiffness changes occurred after throwing and were related to the motion analysis, but the regions in which stiffness occurred varied between pitchers and position players.

## 1. Introduction

Approximately 30% of baseball players experience shoulder pain when throwing [1,2], as a result of an accumulation of microtrauma from highly repetitive overhand throwing [3]. In the late-cocking phase, the pitcher moves the arm backward so that the long axis of the forearm is approximately parallel to the horizontal plane. The range of motion of external rotation of the shoulder joint, which is said to be approximately 100°, exceeds 160° during the late-cocking phase, and the acceleration to internal rotation is more than 6000° per second [4]. In the follow-through phase, the arm moves forward of the body, the elbow joint is extended, and the shoulder joint is internally rotated, and thus a traction force of approximately 108% of the body weight acts on the posterior capsule [5]. As a result of repetitive cocking movements and traction forces during follow-through, the posterior capsule and muscles become thicker and stiffer [6,7]. This change in the stiffness around the posterior shoulder joint can cause glenohumeral internal rotation deficit, which contributes to increased internal shoulder joint pressure and the development of internal impingement. This could result in rotator cuff and labrum injuries, which are organic diseases of the shoulder caused by the throwing motion [6,8]. A subset of 43 baseball and softball players across four studies yielded a 79% rate of return to sports; however, only 38% returned to the same level of play or higher [9], these injuries should be prevented. In fact, these changes in stiffness around the shoulder muscles are one of the major factors in the onset and exacerbation of throwing disorders. Pitchers are injured 3.6–5.8 times more frequently than position players [10]. A quantitative evaluation of the changes in stiffness around the shoulder muscles will be a useful diagnostic indicator for understanding the causes of a disabled throwing shoulder and estimating the risk of recurrence of that. However, no studies have examined the relationship between throwing form and the differences between pitchers and position players. Additionally, no method has been established to evaluate the changes using images.

Several recent studies using ultrasonic shear wave elastography (SWE) have been reported. This technique uses acoustic radiation force produced via a transducer to create shear waves, and the velocity of the waves is measured using ultrasonography as they travel through tissue. The stiffness of skeletal muscles can be measured using SWE [11,12,13], which is useful in predicting retears after rotator cuff repair surgery [14,15], evaluating changes in stiffness of the rotator cuff and joint capsule in a frozen shoulder [16], and evaluating the healing process of muscle injury [17]. In the disabled throwing shoulder, SWE has also been used to analyze the sequential changes in muscle elasticity in the shoulder girdle before and after pitching [18] and to evaluate the elasticity of the posterior shoulder muscles after stretching [19].

We hypothesized that the stiffness of the posterior shoulder muscles increases after throwing, these muscles become stiffer relative to the speed of the arm action, and the muscle strengths of these decrease. The aims of this study were (1) to quantitatively evaluate the change in stiffness using SWE between pre- and post-throwing and to identify the areas where change occurs; (2) to quantitatively evaluate muscle strength in the shoulder region between pre- and post-throwing; and (3) to examine the relationship of change in stiffness to the analysis of the throwing motion. Identifying the relationship between muscle stiffness and muscle strength and the relationship between muscle stiffness and throwing motion analysis by throwing may allow for stretching and training interventions for those muscles, which can result in the prevention of a disabled throwing shoulder.

## 2. Materials and Methods

### 2.1. Participants

**Figure 1:**
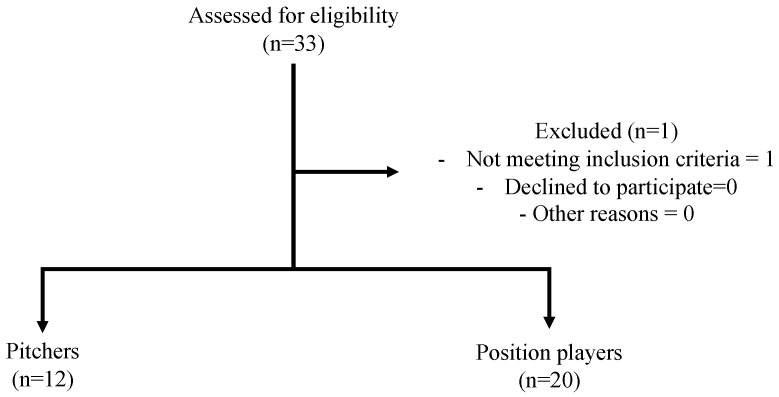
CONSORT flow diagram depicting the progression of participants through a clinical trial.

This study was a descriptive laboratory study approved by the Ethics Committee of Juntendo University Urayasu Hospital (protocol code U20-0069 and date of approval on 1 April 2021), and written informed consent was obtained from all participants. The current male college baseball players from one university volunteered to participate in the study from March 2022 to February 2023. The inclusion criteria were that the participant had at least 5 years of baseball experience and was currently a college baseball player. The exclusion criteria were (1) a history of shoulder and elbow injury requiring them to miss playing time, (2) any pain in the upper extremities at the time of the examination, and (3) a throwing motion that was not overhead. Figure 1 shows the CONSORT flow diagram.

### 2.2. Evaluation of SWE Ultrasound

**Figure 2:**
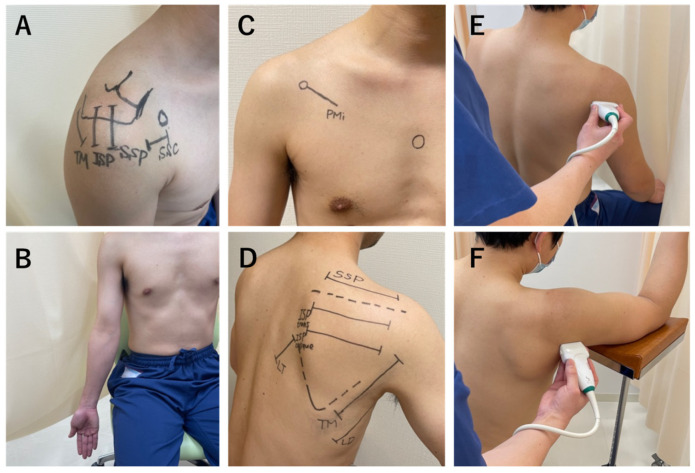
Measurement points for the shear wave elastography (SWE) examinations. (**A**) Probe placement for each tendon; SSC, subscapularis; SSP, supraspinatus; ISP, infraspinatus; TM, teres minor. (**B**) Posture and site for measurement of SSC tendon stiffness. The participant was scanned along the tendon with the upper extremity in a 45° external rotation position with the upper extremity drooped. (**C**,**D**) Probe placement for each muscle; PMi, pectoralis minor; SSP, supraspinatus; ISP trans, the transverse part of the infraspinatus; ISP oblique, the oblique part of the infraspinatus; TM, teres minor; LD, latissimus dorsi; LT, lower trapezius. Positioning of the participants during SWE examination of the posterior capsule (**E**) and the posteroinferior capsule (**F**).

**Figure 3:**
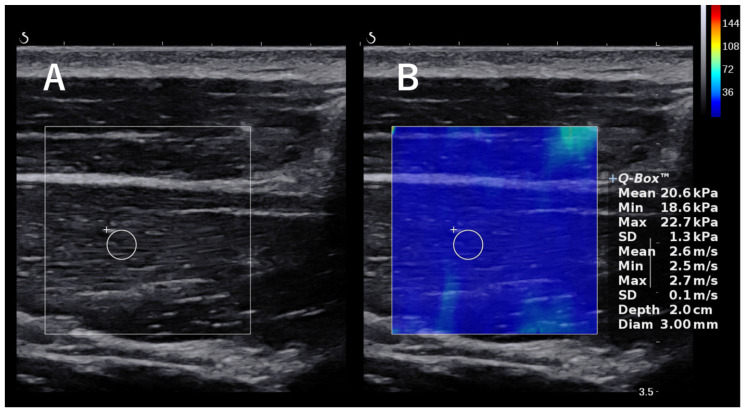
The images of shear wave elastography. The circle in the figure is the region of interest. (**A**) B-mode ultrasound image showing the supraspinatus muscle. (**B**) B-mode image with overlaid elastogram. The circular region of interest was set near the central part of the muscle.

SWE was measured using an Aixplorer (Supersonic Imagine, Aix-en-Provence, France). The examinations were performed by a single shoulder surgeon with 6 years of experience who is experienced with the ultrasound technique. Subjects were examined using SWE to assess the elasticity of muscles and associated components in the shoulder region, including the supraspinatus (SSP) tendon, infraspinatus (ISP) tendon, subscapularis (SSC) tendon, teres minor (TM) tendon, SSP muscle, ISP muscle transverse part, ISP oblique part, TM muscle, lower trapezius (LT) muscle, latissimus dorsi (LD), pectoralis minor (PMi) muscle, posterior capsule, and posteroinferior capsule. When measuring the SSP tendon, ISP tendon, and TM tendons, the participants were scanned in a seated position with the throwing arm relaxed (forearm resting on the ipsilateral thigh) along the tendon. When measuring the SSC tendon, the participants were scanned along the tendon with the upper extremity in a 45° external rotation position with the upper extremity drooped (Figure 2B). When the probe was applied to the supraspinatus fossa, the trapezius muscle was identified in the superficial layer and the SSP muscle was observed beneath it. The ISP transverse and oblique parts were scanned along the muscle fibers positioned 1 cm and 2 cm from the center of the scapular spine, respectively [20]. For the low trapezius muscle, the lateral edge of the transducer was placed 5 cm below the root of the spine of the scapula, and the body of the transducer was inclined at approximately 55° to appear on the long axis of the muscle, as described in a previous study [21]. The LD was scanned by placing the probe parallel to the muscle fibers at three fingerbreadths distal to and along the posterior axillary fold [22]. When the probe was placed medial to the coracoid process, the PMi muscle, which is attached to the coracoid process, was observed beneath the pectoralis major muscle. The measurement site of the PMi was defined as the midpoint between the coracoid process and the fourth rib and sternum junction [23]. The location of the transducer was adjusted to clearly visualize the humeral head, glenoid rim, labrum, and ISP, and the elasticity of the posterior capsule beneath the ISP was measured 5 mm lateral to the edge of the labrum. The location of the transducer was adjusted to clearly visualize the humeral head, glenoid rim, labrum, and TM muscle, and the elasticity of the posteroinferior capsule was measured in the same manner as the measurements of the posterior capsule (i.e., at a location 5 mm lateral to the edge of the labrum) [24]. SWE measurements of the SSP tendons, ISP tendons, TM tendons, and SSC tendons were taken just above the medial border of the tendon attachment. Figure 2 shows the probe placement for each muscle; all muscles were scanned along the long axis, displaying B-mode images. A color-coded box showing the shear elastic modulus was superimposed on the B-mode ultrasound image, and the circular region of interest was set near the central part of the muscle (Figure 3). We performed each measurement twice and used the mean of the two values for the analysis.

The intrarater and interrater reliability of SWE were assessed. The SWE value was measured independently by two investigators who were experienced orthopedic surgeons to assess interrater reliability. The measurements were repeated twice by each investigator to assess intrarater reliability.

### 2.3. Muscle Strength Measurement

Two investigators assessed muscle strength using the Dynamometer IsoForceControl EVO2 (Herkules Kunststoff AG, Oberburg, Switzerland) to measure abduction in the scapular plane in a standing position, internal and external rotation at 0° abduction in a standing position, internal and external rotation at 90° abduction in a supine position, and internal and external rotation at 90° flexion in a supine position. The elbow was in full extension when measuring abduction of the scapular plane, but otherwise the elbow flexion was 90 degrees. This dynamometer provides an isometric measurement of muscle strength over a selectable time of 5 s. The participants were instructed not to apply force to the trunk or other parts of the body during the measurement. The maximum value of the muscle strength when force was applied for 5 s was used for analysis.

### 2.4. Throwing Motion Analysis

**Figure 4:**
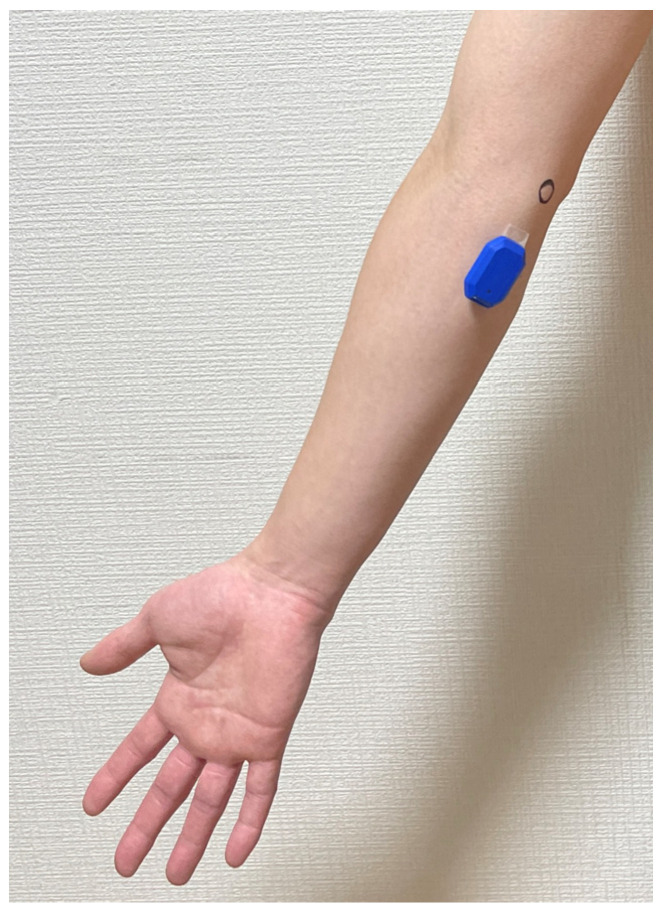
Placement of the inertial measurement unit sensor using double-sided adhesive instead of the provided sleeve. The circle in the figure is the medial epicondyle of the humerus.

Recently, inertial measurement unit (IMU) technology has allowed for the evaluation of throwing kinematics and kinetics. The PULSE Throw Workload Monitor (Driveline Baseball, Kent, WA, USA) IMU with a triaxial accelerometer and triaxial gyroscope was used to measure elbow varus torque and arm slot, arm speed, and arm rotation. Previous research has shown that the IMU measures correlate well with laboratory measures [25] and provide precise and reproducible data [26]. Participants were instructed in how to wear the sleeve correctly, with the sensor on the medial side of the forearm, roughly two finger widths distal to the medial epicondyle of the humerus (Figure 4). The mean of the four items was used in the analysis.

### 2.5. Testing Procedures

The SWE value and muscle strength on the throwing side were measured before and immediately after throwing. The measurements were initially taken before the participants performed any warm-up exercises or the throwing program. After jogging, generalized full-body stretching, and a warm-up, all participants were assured that they could throw to the best of their ability. After confirming that the full intensity was requested, all participants began throwing 18.44 m. The participants performed 20 throws at full intensity, and the measurements of SWE and muscle strength were repeated within 30 min of concluding the throwing.

### 2.6. Statistical Analysis

Data are expressed as means ± standard deviation and were evaluated for all players, pitchers, and position players. We evaluated the intrarater and interrater reliability using the intraclass correlation coefficient (ICC). The Wilcoxon signed-rank test was performed to compare SWE values and muscle strength before throwing and after throwing. Comparisons between the pitcher and position player groups in each of the throwing motion analysis items were analyzed using Mann–Whitney’s U test. The SWE ratio was calculated as (SWE after throwing/SWE before throwing), and the correlation between each item of the throwing motion analysis and SWE ratio was examined by calculating Spearman’s rank correlation coefficient. Statistical significance for all tests was set at *p* < 0.05. All analyses were performed with GraphPad Prism (version 10, San Diego, CA, USA).

## 3. Results

### 3.1. Participates

There were 32 male college baseball players who met these exclusion and inclusion criteria. Participants’ characteristics are shown in Table 1. Their mean age was 20.5 ± 1.6 years (range, 19–24 years); 12 were pitchers; and 20 were position players. The mean length of their baseball career was 10.1 ± 2.9 years (range, 5–15 years).

### 3.2. Reliability of Ultrasound Findings on Stiffness of Shoulder Muscles and Tendons

Table 2 shows the reliability values for the SWE measurements of the shoulder muscles and tendons. The intrarater ICC was 0.772 to 0.931 and the interrater ICC was 0.718 to 0.925, indicating good reliability.

### 3.3. Evaluation of SWE Values and Muscle Strength

Mean SWE values and muscle strength at two time points for 13 items around the shoulder joint are shown (Table 3: all-player group, Table 4: pitcher group, and Table 5: position player group).

Overall, the SWE values of the TM and LD muscles after throwing were significantly higher than those before throwing, whereas the SWE value of the PMi muscle was significantly lower than that before throwing. In the pitcher group, the SWE values of the TM and LD muscles were significantly higher after throwing. In the position player group, the SWE value of the TM muscle was significantly higher after throwing, whereas that of the PMi was significantly lower. No significant difference was found in muscle strengths after throwing in any of the groups in this study.

### 3.4. Correlation between SWE Ratio and Each Item in the Throwing Motion Analysis

**Figure 5:**
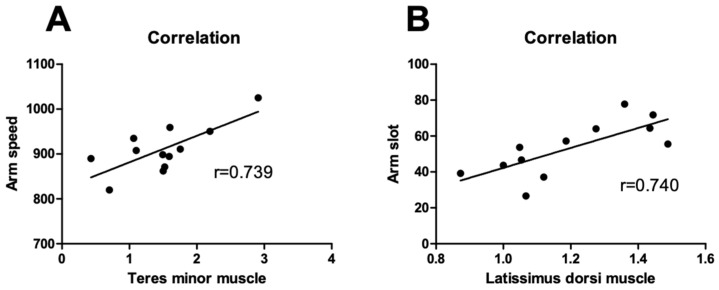
Correlations between throwing motion analysis and SWE ratios in the pitcher group. There was a positive correlation between arm speed and the SWE ratio of the teres minor muscle (**A**) and a positive correlation between arm slot and the SWE ratio of the latissimus dorsi muscle (**B**).

Figure 5 shows the correlation between the throwing motion analysis and SWE ratios in the pitcher group. A positive correlation was found between arm speed and the SWE ratio of the TM muscle (r = 0.739). A positive correlation was also found between the arm slot and the SWE of the LD muscle (r = 0.740). No significant correlations between the throwing motion analysis and SWE ratios were observed in the overall and position player groups.

Table 6 shows the comparative results of the throwing motion analysis between pitchers and position players. There were no significant differences between pitchers and position players in the four items measured by the IMU.

## 4. Discussion

In this study, the SWE values of the TM and LD muscles increased after throwing in the all-player group, whereas the SWE values of the PMi muscles decreased. A previous study of electromyograms of the shoulder muscles during pitching in 56 pitchers reported that the range of motion of external rotation of the shoulder joint increased during the cocking phase due to concentric contraction of the TM muscle, that concentric contraction of the LD muscle during the acceleration phase produced an angular velocity of internal rotation of approximately 6500° per second, and that the posterior muscles such as the TM and LD muscles contracted eccentrically to decelerate the arm speed during the deceleration phase [27]. In this study, the stiffness of the TM muscle might increase as a result of concentric contraction during the cocking phase and eccentric contraction during the deceleration phase. Indeed, a positive correlation was found between arm speed and SWE values of the TM muscles, although only in the pitcher group. The relationship between SWE and throwing motion analysis shows that as the arm speed increases, a large eccentric contraction is required to decelerate the arm, causing a marked increase in stiffness. The stiffness of the LD muscle also might increase as a result of concentric contraction during the acceleration phase and eccentric contraction during the deceleration phase. A positive correlation was also found between arm slot and the SWE values of the LD muscle, although only in the pitcher group. As the increase in the arm slot became greater, the LD muscle length increased. This shows that eccentric contraction with greater muscle length increased the stiffness of the LD muscles. During the late-cocking phase, when the humerus has the maximum external rotation, the scapula is in upward rotation, with a posterior tilt and external rotation [28], at which time the PMi muscle is extended. Many previous studies have reported that eccentric contraction acutely increases muscle stiffness [29,30,31]. In contrast, some reports suggest that low-load eccentric contraction may result in an acute decrease in muscle stiffness [32]. Recent studies have also demonstrated that muscle stiffness is acutely decreased by repeated muscle contractions [33,34,35]. In the present study, the decrease in stiffness in the PMi muscle after throwing may be due to a low throwing load or to a temporary response caused by repetitive muscle contractions.

In the pitcher group, the stiffness of the TM and LD muscles increased after throwing. In the position player group, the stiffness of the TM muscle increased, but the stiffness of the PMi muscle decreased after throwing. The throwing motion analysis showed no difference in throwing form between the pitcher group and the position player group. This suggests that there may be differences in the areas where stiffness changes occur even with the same throwing form. Previous studies have reported different stiffnesses in the posterior shoulder muscles between pitchers and position players [36]. These positional characteristics may influence the difference in stiffness that occurs between pitchers and position players. Further study should be conducted to evaluate differences in stiffness according to position.

After the subjects had thrown 20 balls as hard as possible, there were changes in the SWE value in the TM, LD, and PMi muscles but no changes in muscle strength. According to a previous study that investigated changes in muscle strength in 10 members of a university baseball team after throwing 60 pitches of approximately 18 m as hard as they could, external rotator muscle strength decreased immediately after pitching and returned to normal on the third day after pitching [37]. The small number of throws in the present study did not result in muscle strength changes, suggesting that muscle stiffness changes may occur before muscle strength changes occur. Muscle strength changes during the throwing motion can lead to compensatory movements that can injure the athlete. Changes caused by muscle fatigue from the throwing motion are associated with kinematic changes that decrease tissue tolerance and increase the risk of injury [38]. Assessing muscle stiffness may help to determine the muscles that may experience early muscle weakness, which may lead to the prevention of disability and therapeutic intervention.

A previous study reported an increase in stiffness in all muscles measured (SSP, ISP, middle trapezius, LT, serratus anterior, and rhomboid muscles) after 100 pitches [18]. And the mean elasticity values of the infraspinatus and the lower trapezius muscle remained elevated 24 h after pitching. This suggests that the higher load applied to the shoulder in the throwing motion increases the stiffness of the more posterior shoulder muscle groups. In this study, changes in muscle stiffness occurred immediately after 20 throwing movements. The fact that the mean elasticity values changed even with a small number of pitches suggests that pitching motion had some effect on these muscles, causing a more specific change in stiffness. In other words, this leads to the identification of muscles that are more susceptible to the effects of throwing. In the present study, the stiffness of the TM muscles increased in pitchers, position players, and all players, regardless of position. No previous studies using SWE have focused on the TM muscle, but this was the muscle that stiffened after throwing in all athletes in the present study. Cross-body stretching, performed with 90° flexion in the throwing side-lying position with the humerus of the throwing side moved into internal rotation using the arm of the non-throwing side, has been reported to reduce the stiffness of the TM muscle [19]. It is suggested that this type of stretching might help prevent a disabled throwing shoulder.

This study identified muscles that are subjected to early loading in throwing and revealed that there are differences depending on position. This fact reveals the muscles to focus on, such as training and stretching, depending on position, and may prevent injury from throwing. It was also found that muscle stiffness changes were observed before muscle strength changes. It was suggested that measuring muscle stiffness may be one indicator to prevent large loading in rehabilitation and for the prevention of a disabled throwing shoulder.

This study had some limitations. First, the study focused on a single college baseball team and evaluated a relatively small sample size because players with current shoulder and elbow pain and players with a non-overthrow form of motion were excluded. Second, the IMU used for the throwing motion analysis is very simple and easy to use, but its measurement items are the forearm angle and shoulder joint external rotation angle from the ground. In other words, it is the sum of multiple joint ranges of motion, including the pelvis and vertebrae. Although the throwing motion is a whole-body movement, it is preferable to separately evaluate the movement between each joint segment for muscle evaluation. Previous research had shown that the IMU measures correlate well with laboratory measures and provide precise and reproducible data. But recently study demonstrated that the values provided by the IMU should not be considered equivalent to those generated by the gold standard of marker-based motion capture [39]. It may be difficult to use the IMU to evaluate throws. Future research should be conducted to investigate the effect of the movement of each segment on stiffness. The third point is that the results of this study indicate which phase caused the stiffness change, but this is only a guess since the electromyogram was not actually measured. The development of a new device that can capture the characteristics of each phase is desired in the future. Fourth, we did not investigate any associations with pain. Future investigation of the correlation between pain, throwing motion analysis, and muscle stiffness may lead to a better understanding of the pathophysiology of a disabled throwing shoulder.

## 5. Conclusions

The SWE values of the TM and LD muscles increased in the pitcher group, whereas the SWE values of the TM muscle increased and those of the PMi muscles decreased in the position player group. Positive correlations were found between arm speed and the TM muscles and between arm slot and the LD muscle in the pitcher group.

## Figures and Tables

**Table 1:** Participants’ characteristics.

	All	Pitchers	Position Players
Number	32	12	20
Age (yr)	20.5 ± 1.6	21.0 ± 1.6	20.1 ± 1.5
Height (cm)	172.3 ± 5.8	172.1 ± 6.2	172.3 ± 5.9
Weight (kg)	71.5 ± 4.7	72.1 ± 6.4	70.8 ± 6.4
Baseball career (yr)	10.1 ± 2.9	10.5 ± 3.5	9.75 ± 2.5
Dominant hand	Right-29, Left-3	Right-10, Left-2	Right-19, Left-1

**Table 2:** Reliability of ultrasound measurements for muscle elasticity.

	ICC for Intrarater	ICC for Interrater
Measuring Section of SWE	Reliability	Reliability
Supraspinatus tendon	0.825	0.829
Infraspinatus tendon	0.806	0.797
Teres minor tendon	0.814	0.846
Subscapularis tendon	0.772	0.781
Supraspinatus muscle	0.861	0.856
Infraspinatus muscle (transverse)	0.821	0.780
Infraspinatus muscle (oblique)	0.824	0.821
Teres minor muscle	0.826	0.813
Lower trapezius muscle	0.829	0.718
Latissimus dorsi muscle	0.931	0.925
Pectoralis minor muscle	0.874	0.853
Posterior capsule	0.783	0.744
Posteroinferior capsule	0.785	0.780

**Table 3:** Mean elasticity values and muscle strength values at 2 time points in the all-player group. Bolded *p* values indicate a statistically significant difference (*p* < 0.05).

**Measuring Section (Elasticity)**	**Before Throwing (kPa)**	**After Throwing (kPa)**	***p* Values**
Supraspinatus tendon	364.0 ± 122.1	387.1 ± 116.0	0.472
Infraspinatus tendon	423.8 ± 118.3	415.3 ± 132.0	0.993
Teres minor tendon	347.1 ± 128.5	367.9 ± 149.6	0.250
Subscapularis tendon	404.7 ± 93.7	424.3 ± 87.9	0.299
Supraspinatus muscle	26.2 ± 11.0	28.2 ± 14.4	0.307
Infraspinatus muscle (transverse)	17.3 ± 4.2	18.4 ± 5.4	0.250
Infraspinatus muscle (oblique)	18.1 ± 5.4	20.0 ± 8.5	0.688
Teres minor muscle	20.7 ± 6.3	29.6 ± 11.1	**0.003**
Lower trapezius muscle	10.0 ± 3.1	11.4 ± 3.7	0.116
Latissimus dorsi muscle	19.7 ± 6.1	20.8 ± 7.6	**0.007**
Pectoralis minor muscle	16.0 ± 5.3	12.8 ± 4.8	**0.006**
Posterior capsule	77.3 ± 73.8	114.5 ± 117.9	0.074
Posteroinferior capsule	94.5 ± 68.0	102.7 ± 91.8	0.715
**Measuring Section (Muscle Strength)**	**Before Throwing (N)**	**After Throwing (N)**	***p* Values**
Abduction	124.9 ± 48.9	129.8 ± 39.7	0.345
Internal rotation abduction 0°	84.9 ± 19.8	87.6 ± 14.7	0.200
External rotation abduction 0°	69.4 ± 14.2	69.5 ± 13.9	0.918
Internal rotation abduction 90°	82.9 ± 14.6	84.8 ± 13.6	0.466
External rotation abduction 90°	83.8 ± 21.5	80.6 ± 17.7	0.326
Internal rotation elevation 90°	78.1 ± 20.4	77.5 ± 15.5	0.794
External rotation elevation 90°	60.1 ± 16.8	57.6 ± 13.2	0.270

**Table 4:** Mean elasticity values and muscle strength values at 2 time points in the pitcher group. Bolded *p* values indicate a statistically significant difference (*p* < 0.05).

**Measuring Section (Elasticity)**	**Before Throwing (kPa)**	**After Throwing (kPa)**	***p* Values**
Supraspinatus tendon	372.3 ± 134.4	343.3 ± 125.1	0.677
Infraspinatus tendon	402.3 ± 128.1	387.5 ± 147.6	1.000
Teres minor tendon	343.1 ± 133.1	323.0 ± 155.9	0.733
Subscapularis tendon	387.7 ± 72.4	395.0 ± 98.1	0.910
Supraspinatus muscle	28.8 ± 15.0	30.0 ± 19.1	1.000
Infraspinatus muscle (transverse)	17.7 ± 5.2	18.2 ± 6.7	0.530
Infraspinatus muscle (oblique)	20.2 ± 6.5	17.4 ± 5.3	0.176
Teres minor muscle	22.0 ± 6.0	30.9 ± 13.0	**0.043**
Lower trapezius muscle	11.0 ± 3.8	12.9 ± 4.1	0.064
Latissimus dorsi muscle	18.9 ± 4.0	22.3 ± 5.8	**0.014**
Pectoralis minor muscle	15.7 ± 4.9	13.1 ± 4.9	0.224
Posterior capsule	68.0 ± 48.2	74.2 ± 52.0	0.424
Posteroinferior capsule	79.7 ± 55.4	95.8 ± 94.5	0.910
**Measuring Section (Muscle Strength)**	**Before Throwing (N)**	**After Throwing (N)**	***p* Values**
Abduction	112.7 ± 37.2	113.4 ± 26.9	0.845
Internal rotation abduction 0°	80.5 ± 13.3	83.4 ± 14.6	0.326
External rotation abduction 0°	69.4 ± 16.8	70.3 ± 14.0	0.505
Internal rotation abduction 90°	84.8 ± 11.5	84.8 ± 10.8	0.970
External rotation abduction 90°	89.0 ± 19.0	80.8 ± 18.1	0.077
Internal rotation elevation 90°	78.7 ± 19.4	74.2 ± 12.1	0.233
External rotation elevation 90°	56.3 ± 15.2	57.7 ± 9.4	0.233

**Table 5:** Mean elasticity values and muscle strength values at 2 time points in the position player group. Bolded *p* values indicate a statistically significant difference (*p* < 0.05).

**Measuring Section (Elasticity)**	**Before Throwing (kPa)**	**After Throwing (kPa)**	***p* Values**
Supraspinatus tendon	359.0 ± 117.4	413.3 ± 104.6	0.185
Infraspinatus tendon	436.8 ± 113.4	432.0 ± 122.6	0.955
Teres minor tendon	349.6 ± 129.0	394.9 ± 142.8	0.255
Subscapularis tendon	414.8 ± 104.8	442.0 ± 78.5	0.097
Supraspinatus muscle	24.6 ± 7.8	27.1 ± 11.1	0.218
Infraspinatus muscle (transverse)	17.0 ± 3.7	18.5 ± 4.7	0.341
Infraspinatus muscle (oblique)	16.8 ± 4.4	21.5 ± 9.7	0.151
Teres minor muscle	19.9 ± 6.5	28.8 ± 10.1	**0.002**
Lower trapezius muscle	9.3 ± 2.5	10.5 ± 3.2	0.641
Latissimus dorsi muscle	20.2 ± 7.1	20.0 ± 8.5	0.140
Pectoralis minor muscle	16.1 ± 5.6	12.6 ± 4.8	**0.016**
Posterior capsule	82.9 ± 86.3	138.7 ± 139.5	0.131
Posteroinferior capsule	103.4 ± 74.4	106.8 ± 92.4	0.867
**Measuring Section (Muscle Strength)**	**Before Throwing (N)**	**After Throwing (N)**	***p* Values**
Abduction	132.2 ± 54.3	139.6 ± 43.4	0.341
Internal rotation abduction 0°	87.5 ± 22.8	90.2 ± 14.5	0.401
External rotation abduction 0°	69.3 ± 12.8	69.0 ± 14.2	0.332
Internal rotation abduction 90°	81.8 ± 16.4	84.8 ± 15.3	0.970
External rotation abduction 90°	80.5 ± 22.7	80.5 ± 18.0	0.837
Internal rotation elevation 90°	77.7 ± 21.4	79.5 ± 17.2	0.638
External rotation elevation 90°	62.4 ± 17.7	57.6 ± 15.3	0.117

**Table 6:** Items of throwing motion analysis measured by PULSE throw workload monitor IMU.

Measuring Section	Pitchers	Position Players	*p* Values
Elbow torque (Nm)	37.7 ± 6.7	34.4 ± 8.3	0.321
Arm slot (deg)	53.1 ± 15.1	61.4 ± 11.9	0.179
Arm speed (RPM)	910.3 ± 52.8	876.0 ± 90.0	0.251
Shoulder rotation (deg)	149.2 ± 18.3	145.7 ± 12.4	0.340

## Data Availability

Data are contained within the article.
